# Interlayer Nano‐Dots Induced High‐Rate Supercapacitors

**DOI:** 10.1002/advs.202301398

**Published:** 2023-06-04

**Authors:** Chunyan Li, Xinkun Wang, Dongge Ma, Yan Yan, Pengwei Huo, Qingjun Yang

**Affiliations:** ^1^ Research Center of Fluid Machinery Engineering and Technology Jiangsu University Zhenjiang 212013 P. R. China; ^2^ School of Chemistry and Chemical Engineering Jiangsu University Zhenjiang 212013 P. R. China; ^3^ Department of Chemistry College of Chemistry and Materials Engineering Beijing Technology and Business University Beijing 100048 P. R. China

**Keywords:** CdS nano‐dots, interlayer redox reaction, layered double hydroxides, layer spacing regulation, supercapacitors

## Abstract

The fast OH^−^ transfer between hydroxide layers is the key to enhancing the charge storage efficiency of layered double hydroxides (LDH)‐based supercapacitors (SCs). Constructing interlayer reactive sites in LDH is much expected but still a huge challenge. In this work, CdS nano‐dots (NDs) are introduced to interlayers of ultra‐thin NiFe‐LDH (denoted CdS_inter._‐NiFe‐LDH), promoting the interlayer ions flow for higher redox activity. The excellent performance is not only due to the enlarged layer spacing (from 0.70 to 0.81 nm) but also stems from anchored interlayer reactive units and the undamaged original layered structure of LDH, which contribute to the improvement of OH^−^ diffusion coefficient (1.6 × 10^−8^ cm^2^ s^−1^) and electrochemical active area (601 mF cm^−2^) better than that of CdS NDs on the surface of NiFe‐LDH (2.1 × 10^−9^ cm^2^ s^−1^ and 350 mF cm^−2^). The champion CdS_inter._‐NiFe‐LDH electrode displays high capacitance of 3330.0 F g^−1^ at 1 A g^−1^ and excellent retention capacitance of 90.9% at 10 A g^−1^, which is better than the NiFe‐LDH with CdS NDs on the surface (1966.6 F g^−1^). Moreover, the assembled     asymmetric SCs (ASC) device demonstrate an outstanding energy density/power density (121.56 Wh kg^−1^/754.5 W kg^−1^).

## Graphical abstract

Constructing interlayer active sites is the key to improving the charge storage efficiency of LDH‐based supercapacitors. In this work, via a feasible in situ growth method, CdS nanodots are introduced between NiFe‐LDH layers, improving the OH^−^ flow and redox activity, which exhibits a record high capacitance of 3330.0 F g^−1^.

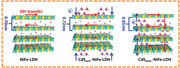

## 1 Introduction

**Scheme 1:**
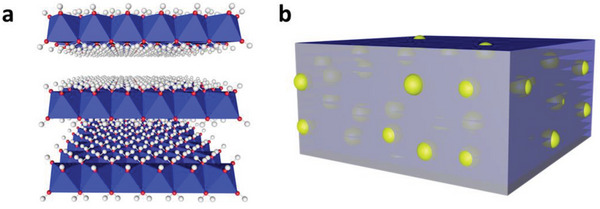
a) Schematic diagram of the interlayer structure of LDH, b) the schematic diagram of the designed blueberry melaleuca cake structure.

The rapid consumption of fossil fuels and subsequent environmental problems have prompted the progress of sustainable energy conversion and storage.^[^ ^1^, ^2^ ^]^ Supercapacitors (SCs) provide a promising energy storage solution route on account of the first‐class power density and long cycling performances.^[^ ^3^, ^4^ ^]^ Typically, high‐performance electrode materials for SCs should possess a large specific surface area and rapid transfer of ions/electrons at the surface of electrode to facilitate the rapid redox reactions and increase the capacitance. Layered double hydroxides (LDH) are thus widely used in SCs due to their unique 2D hierarchical layered structure, which can increase the reactive area with electrolytes. However, the easy aggregation of LDH restricts the diffusion of electrolyte ions in the electrode, thus constructing regular morphology of LDH nanosheets with nano‐scale thickness and reduced transverse size has become the premise of subsequent research to expose more active sites.^[^ ^5^ ^]^ Pioneering works demonstrate that the morphology and lattice structure of NiFe‐LDH are affected by different synthesis methods.^[^ ^6^, ^7^ ^]^ Nevertheless, the interlayer of LDH in the lattice is wholly exposed to hydroxyl (—OH) groups (**Scheme** **1**a), which extremely limit the number of active sites required for rapid redox reaction and OH^−^ transfer on the inner surface.^[^ ^8^, ^9^, ^10^ ^]^ It is an effective strategy to expose metal active units on the inner surface by building defective sites.^[^ ^11^, ^12^, ^13^ ^]^ However, owing to the narrow interlayer spacing of LDH, the increased reactive centers from defective sites are limited. More importantly, excessive defects destroy the integrity of the LDH bulk lattice thereby reducing the conductivity of LDH.^[^ ^14^, ^15^ ^]^ Figuring out a general strategy for constructing sufficient reactive sites on the inert hydroxide interlayer is crucial to realizing high‐rate LDH‐based SCs devices.

Recently, the intercalation of metal ions (e.g., Na^+^, K^+,^ Ca^2+^, Mg^2+^, and Zn^2+^) into interlayers of LDH by electrochemical cycling has been reported,^[^ ^16^ ^]^ which utilizes intercalated metal ions as reaction sites between each layers to improve electrode performances. However, stably anchoring multiple metal ions on hydroxyl‐exposed interlayer is very difficult and unfavorable to the fast OH^−^ transfer in alkaline electrolytes.^[^ ^17^, ^18^ ^]^ Moreover, after several charging/discharging cycles, metal ions can further intercalate into the layered lattice of LDH with less metal coordination by forming an inert passivation layer,^[^ ^16^, ^19^, ^20^ ^]^ which inevitably prevents the interlayer redox reaction. As an alternative strategy, constructing hybrid hetero‐junctions by in situ modification of interlayer or surface of LDH was proposed.^[^ ^21^, ^22^, ^23^ ^]^ In our previous work, by forming Co_9_S_8_ Quantum‐Dots on NiCoZn‐LDH through the in situ vulcanization of exposed Co sites, 1.8 times increased capacitance was obtained.^[^ ^24^ ^]^ We argue that the in situ deposition of transition metal‐based NDs between LDH interlayers in a melaleuca cake structure (Scheme 1b) will break through the bottleneck that the interlayer surface of LDH is inert to redox reaction, further facilitating OH^−^ transfer between interlayers and improving the rate and capacitance performances of LDH‐based SCs devices.

Herein, via introducing CdS NDs in the bulk of LDH lattice based on a simple electrochemical cycling and low‐temperature vulcanization strategy, the capacitance of NiFe‐LDH with a melaleuca cake structure (denoted CdS_inter._‐NiFe‐LDH) is 1.7 times of the NiFe‐LDH modified with CdS NDs on the surface (denoted CdS_surf._‐NiFe‐LDH) and 2.6 times of the NiFe‐LDH. The prominent performance is caused by the rapid OH^−^ migration and the increased NDs active sites anchored inside the intercalation of LDH. Moreover, the asymmetric SCs assembled with CdS_inter._‐NiFe‐LDH as anode and ZnCo‐modified porous carbon (ZnCo‐PC) as cathode achieve a remarkable energy density of 121.56 Wh kg^−1^.

## 2 Results and Discussion

**Figure 1:**
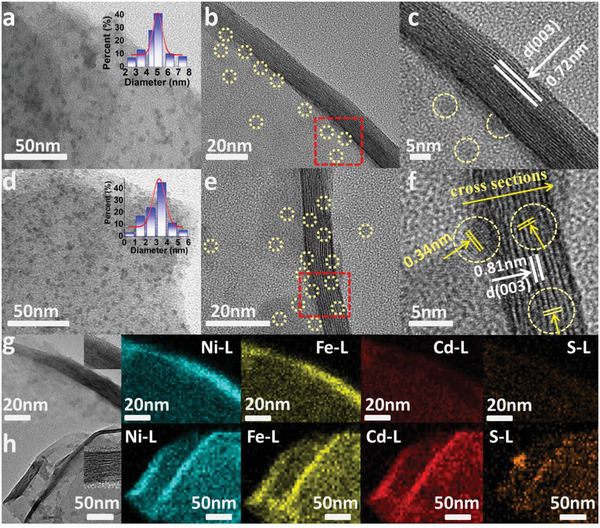
a) The TEM image (the inset is the size distribution of NDs), b) the magnified TEM image, and c) the HRTEM image of CdS_surf._‐NiFe‐LDH. d) The TEM image (the inset is the size distribution of NDs), e) the magnified TEM image, and f) the HRTEM image of CdS_inter._‐NiFe‐LDH; The element mapping of g) CdS_surf._‐NiFe‐LDH and h) CdS_inter._‐NiFe‐LDH; insets of (g) and (h) show cross‐sections of LDH layers.

The CdS_inter._‐NiFe‐LDH electrode was prepared using the stepwise electrochemical intercalation and low‐temperature vulcanization strategy. Cd^2+^ cations were first intercalated into interlayers of NiFe‐LDH by electrochemical cycling (the details of preparation methods are described in Supporting Information). Subsequently, the obtained Cd^2+^/NiFe‐LDH was vulcanized at different temperatures (0–15 °C) to prepare the target CdS_inter._‐NiFe‐LDH electrode. As a comparison, the CdS_surf._‐NiFe‐LDH was obtained by the direct surface Cd^2+^ adsorption and subsequent vulcanization. As shown in Figure S1a (Supporting Information), NiFe‐LDH shows a 2D uniform nanosheet structure. After the CdS modification, CdS_surf._‐NiFe‐LDH and CdS_inter._‐NiFe‐LDH nanosheets exhibit identical morphologies as compared to the pristine NiFe‐LDH, indicating that the loading of CdS NDs does not destroy the morphology of NiFe‐LDH (Figure S1b–d, Supporting Information). As depicted in the transmission electron microscopy (TEM) images, CdS NDs were distributed on the CdS_surf._‐NiFe‐LDH (**Figure** **1**a) and CdS_inter._‐NiFe‐LDH nanosheets (Figure 1d), which are in sharp contrast to the clean surface of pristine NiFe‐LDH (Figure S2a, Supporting Information). According to the statistics of CdS NDs size based on TEM images, the particle size of surface NDs was slightly larger than that of interlayer NDs (3.5–4.3 nm). Through the HRTEM image of LDH layers at the cross section, no CdS NDs could be observed between CdS_surf._‐NiFe‐LDH layers. Instead, they only appeared on both sides of the cross section (Figure 1b). In contrast, CdS NDs in CdS_inter._‐NiFe‐LDH can be observed embedded between (003) plane of NiFe‐LDH at the center of the cross section (Figure 1e). Notably, the lattice d‐spacing distance of the (003) plane for the pristine NiFe‐LDH is ≈7 Å (Figure S2b,c, Supporting Information) and the layer spacing (≈7.2 Å) of (003) plane for the CdS_surf._‐NiFe‐LDH is not significantly changed (Figure 1c). The HRTEM images of CdS_inter._‐NiFe‐LDH show lattice spacing of 3.4 and 8.1 Å, corresponding to (002) plane of CdS and (003) plane of NiFe‐LDH, respectively (Figure 1f). In contrast, the above result provides solid evidence that CdS NDs are embedded in the interlayer and broaden the distance between NiFe‐LDH crystal planes.

In addition, through HAADF‐Mapping scanning, we can clearly distinguish different distribution features of CdS NDs on the upper surface and interlayer cross sections of the two samples. For CdS_surf._‐NiFe‐LDH, the NDs were uniformly distributed in the LDH range, and no obvious enrichment was observed at the cross section (Figure 1g). On the contrary, for CdS_inter._‐NiFe‐LDH, CdS NDs were concentrated with a distinctly brighter edge at the cross section (Figure 1h). The above characterizations fully verify the successful realization of the interlayer and surface CdS NDs modifications. The 3D Atomic Force Microscope (AFM) surface mapping further distinguishes the difference between surface and interlayer NDs loading. For CdS_surf._‐NiFe‐LDH, the mean thickness was ≈6.5 nm, and the aggregation structure of NDs with rough surface (roughness 3–4 nm) can be clearly observed (Figure S3a, Supporting Information). In contrast, the surface of CdS_inter._‐NiFe‐LDH sample was flat, and the LDH monolayer thickness was ≈6.7 nm (Figure S3b, Supporting Information), which is due to the interlayer CdS NDs expand the distance between crystal planes, consistent with the HRTEM observations (Figure 1c,f). By inductively coupled plasma optical emission spectrometry (ICP‐OES) elemental survey, we have enabled the two (CdS_inter._‐NiFe‐LDH and CdS_surf._‐NiFe‐LDH) electrodes to have nearly identical Cd/Ni ratios, thereby directly confirming the performance effects of the nano‐dot loading with respect to the volume phase and surface, excluding other factors (Table S1, Supporting Information).

**Figure 2:**
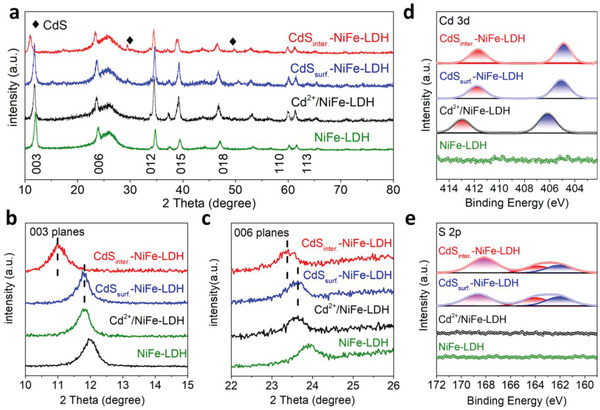
a) XRD graphs of CdS_inter._‐NiFe‐LDH, CdS_surf._‐NiFe‐LDH, Cd^2+^/NiFe‐LDH, and NiFe‐LDH, b) the enlarged XRD patterns for (003) planes, c) the enlarged XRD patterns of (006) planes. The XPS spectra of d) Cd 3d and e) S 2p orbits of CdS_inter._‐NiFe‐LDH, CdS_surf._‐NiFe‐LDH, Cd^2+^/NiFe‐LDH, and NiFe‐LDH.

We further characterized the crystal structures of as‐prepared materials. From X‐ray diffraction (XRD) patterns (**Figure** **2**a), we found that the interlayer adsorption of Cd^2+^ ions and the surface loading of Cd^2+^ ion did not significantly affect the LDH structure with a very slight peak shift of (003) plane. The Scanning electron microscopy‐energy dispersive spectrum (SEM‐EDS) measurement (Figure S4, Supporting Information) proves that Cd^2+^ and Cl^−^ coexisted in the Cd^2+^/NiFe‐LDH sample, suggesting the intercalation of Cd^2+^ ions between LDH layers rather than doping into the lattice. According to literature reports,^[^ ^16^, ^20^, ^25^ ^]^ the increased layer spacing of Cd^2+^/NiFe‐LDH is possibly from both Cd^2+^ and Cl^−^, because cation and anion intercalate simultaneously between layers. On the contrary, the interlayer NDs caused the diffraction peaks of (003) and (006) planes to shift toward the small angle from 12.5˚ to 11.0˚ (Figure 2b) and from 23.92˚ to 23.39˚ (Figure 2c), respectively. According to Bragg's law (2dsin*θ* = n*λ*, n = 1), the interlayer distance of (003) plane of CdS_inter._‐NiFe‐LDH was ≈0.81 nm, and it was better than NiFe‐LDH (0.70 nm) and CdS_surf._‐NiFe‐LDH (0.72 nm), which is consistent with the HRTEM observations (Figure 1c,f) and directly demonstrates that the interlayer CdS NDs enlarge the layer spacing of NiFe‐LDH. This is supposed to facilitate interlayer ion migration and rapid charge–discharge reaction. In addition, factors of the reaction time, reaction temperature, and cycle numbers of Cd^2+^ put a “positive volcano” effect on the size of interlayer space, which finely adjusts the size of interlayer (Figure S5, Supporting Information).

The elemental valence state, composition, and variation in the electronic structure of CdS_inter._‐NiFe‐LDH were directly observed by X‐Ray photoelectron spectroscopy (XPS) measurement. After electrochemical cycles, Cd^2+^/NiFe‐LDH exhibits two spin–orbit doublets in Cd 3d spectra corresponding to Cd 3d_5/2_ and Cd 3d_3/2_ at 406.26 and 412.98 eV, respectively (Figure 2d). After low temperature vulcanization, for CdS_inter._‐NiFe‐LDH and CdS_surf._‐NiFe‐LDH, the diffraction peaks of Cd 3d_5/2_ and Cd 3d_3/2_ were shifted toward lower electron binding energy, suggesting that Cd^2+^ ions were reduced. Correspondingly, the S 2p signal can be observed after the CdS NDs loading (Figure 2e). Notably, the Cd 3d and S 2p spectra were almost identical on two samples, indicating that the interlayer and surface loaded CdS NDs have similar chemical states, and the difference is only reflected in the distinction of spatial location. Furthermore, the binding energy values of Ni 2p and Fe 2p spectra (Figure S6, Supporting Information) exhibit little difference before and after vulcanization, reflecting that the low temperature vulcanization does not change the feature of NiFe‐LDH.

**Figure 3:**
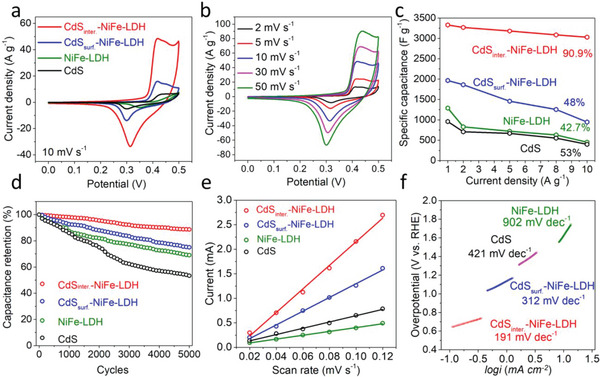
a) the CV curves of CdS_inter._‐NiFe‐LDH, CdS_surf._‐NiFe‐LDH, CdS and NiFe‐LDH at the scanning speed of 10 mV s^−1^, b) the CV curves of CdS_inter._‐NiFe‐LDH at different scanning speed, c) the specific capacitance of CdS_inter._‐NiFe‐LDH, CdS_surf._‐NiFe‐LDH, CdS and NiFe‐LDH varies with various current densities (1–10 A g^−1^), d) the cycle performance of CdS_inter._‐NiFe‐LDH, CdS_surf._‐NiFe‐LDH, CdS and NiFe‐LDH under 5000 times, e) the capacitive current (*I*_DL_) at 0.56 V varies with scanning speed for all electrodes, f) Tafel graphs of CdS_inter._‐NiFe‐LDH, CdS_surf._‐NiFe‐LDH, CdS and NiFe‐LDH.

We believe that the low transfer efficiency of OH^−^ between LDH layers and limited reactive sites on the inert inner surface are the inherent bottlenecks for high‐performance LDH‐based SCs. As a solution, the interlayer loading of CdS NDs can significantly increase the spacing between interlayers and provides rich reaction sites, which endow NiFe‐LDH with both high‐rate performances and large specific capacitance (*C*_s_). First, we performed Cyclic voltammetry (CV) measurements with the same scan rate on samples to explore the influence of interlayer NDs on the capacitance and current response. The CdS_inter._‐NiFe‐LDH exhibits a much larger closed curve area than that of CdS_surf._‐NiFe‐LDH, CdS and NiFe‐LDH electrodes at the scanning speed of 10 mV s^−1^, proving the superior capacitance and kinetic reversibility of CdS_inter._‐NiFe‐LDH (**Figure** **3**a). Moreover, the shapes of CV curve of CdS_inter._‐NiFe‐LDH remain unchanged with the increasing scan rate at the same voltage window, which directly proves its excellent rate performance (Figure 3b). The capacitance performance of CdS_inter._‐NiFe‐LDH and other electrode materials was calculated in detail by the Galvanostatic charge‐discharge (GCD) curves (Figures S7 and S8, Supporting Information). As depicted in Figure 3c, we found that *C*_s_ of CdS_inter._‐NiFe‐LDH was 3330.0 F g^−1^ at 1 A g^−1^, which was 1.7 times of the CdS_surf._‐NiFe‐LDH (1966.6 F g^−1^ at 1 A g^−1^), 2.6 times of NiFe‐LDH (1288.0 F g^−1^ at 1 A g^−1^), and 3.5 times of CdS (960.0 F g^−1^ at 1 A g^−1^) based on Equation 1 (see in Experimental Section). Moreover, the capacitance retention rate of CdS_inter._‐NiFe‐LDH (90.9%) was distinctly superior to CdS_surf._‐NiFe‐LDH (48.0%) at 10 A g^−1^. The *C*_s_ and rate performance were further optimized by adjusting the layer spacing with different reaction temperature, reaction time, and cycle times in Figure S9 (Supporting Information). The stability of the electrode materials was studied by conducting 5000 charge and discharge processes at 1 A g^−1^ (Figure 3d), where CdS_inter._‐NiFe‐LDH sample maintains 88.2% of the initial capacity after 5000 cycles of continuous operation, higher than that of CdS_surf._‐NiFe‐LDH (74.1%) and NiFe‐LDH (68.9%). Despite possessing nearly identical NDs loading and similar chemical states, the SC performance is different due to different positions embedded with NDs and the consequent different width of interlayer spacing. Therefore, we reason that CdS NDs, as introduced reactive sites, can greatly promote the interlayer redox reaction during the charge and discharge process.

Indeed, the electrochemical active surface areas (ECSA) of all electrodes, calculated via CV measurements (Figure S10, Supporting Information) under 0.5–0.62 V versus RHE (based on Equations 2 and 3, seen in Experimental Section), reflect that CdS_inter._‐NiFe‐LDH has the highest ECSA of 601 mF cm^−2^ that is 1.71 times of CdS_surf._‐NiFe‐LDH and 3.75 times of the pristine NiFe‐LDH (Figure 3e), further demonstrating the significant role of interlayer active sites for improving the performance of LDH‐based SCs. In Figure 3f, the Tafel slopes of CdS_inter._‐NiFe‐LDH (191 mV dec^−1^) were much lower than that of CdS_surf._‐NiFe‐LDH (312 mV dec^−1^) and other electrode materials, implying boosted redox kinetics. It is caused by the increased activity of the inert inner surface of LDH from the interlayer NDs, which reduces the over‐potential and accelerate oxidation‐reduction process. As compared to reported LDH‐based electrode materials, our CdS_inter._‐NiFe‐LDH electrode material has superiority in capacitance performance and structural stability (Table S2, Supporting Information). In addition, the content of CdS in CdS_inter._‐NiFe‐LDH was further optimized with different CdCl_2_ solution concentrations (0.1–5 m). The element distribution of different samples is shown in Table S3 (Supporting Information). Through comparisons (Figure S11, Supporting Information), we found that CdS content is not as large as possible. Although CdS nano‐dots can act as OH^−^ active sites, too much CdS will deteriorate the integrity of LDH lattice and thus increase the resistance of electrodes.

**Figure 4:**
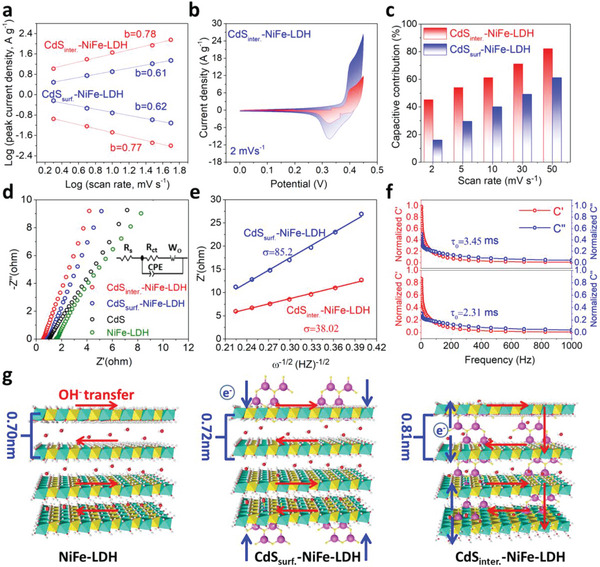
a) The logarithm of anodic and cathodic peak current densities varies with scan rate of CdS_inter._‐NiFe‐LDH and CdS_surf._‐NiFe‐LDH, b) Volta‐metric response for the CdS_inter._‐NiFe‐LDH at 2 mV s^−1^, c) the capacitive contributions of CdS_inter._‐NiFe‐LDH and CdS_surf._‐NiFe‐LDH (2–50 mV s^−1^), d) EIS plots of CdS_inter._‐NiFe‐LDH, CdS_surf._‐NiFe‐LDH, CdS and NiFe‐LDH, e) the Z′ of CdS_inter._‐NiFe‐LDH and CdS_surf._‐NiFe‐LDH varies with *ω* ^−1/2^ at low frequency section, f) normalized *C*′ and *C*′′ of the CdS_inter._‐NiFe‐LDH and CdS_surf._‐NiFe‐LDH as a function of frequency, g) The diffusion mechanism of electron and OH^−^ of NiFe‐LDH, CdS_surf._‐NiFe‐LDH and CdS_inter._‐NiFe‐LDH.

Furthermore, CV measurements in the range of 0–0.45 V that was to exclude the impact of OER were conducted to research the charge storage mechanism and OH^−^ reaction kinetics of CdS_inter._‐NiFe‐LDH electrode. According to Equation 4 (Seen in Experimental Section), the b value was calculated by log (anodic and cathodic peak current densities) and log (scan rate), determining the diffusion (b≤0.5) and capacitive‐controlled behaviors (b≥1) charge storage mechanism.^[^ ^26^, ^27^, ^28^ ^]^ The b values of CdS_inter._‐NiFe‐LDH for anodic and cathodic processes were 0.78 and 0.77, respectively, larger than that of CdS_surf._‐NiFe‐LDH (0.61 and 0.62). Therefore, CdS_inter._‐NiFe‐LDH reveals the mixed charge storage mechanism of diffusion and capacitive‐controlled behaviors, indicating more favorable reaction kinetics and stronger semi‐infinite diffusion controlled reaction (**Figure** **4**a). According to Equation 5 (Seen in Experimental Section), the diffusion‐controlled and capacitive contribution to the total charge storage were surveyed with the curve of I (V)/v^1/2^ versus v^1/2^ at different potentials by Trasatti analysis method (Figure S12a, Supporting Information). The capacitive contribution of the CdS_inter._‐NiFe‐LDH was 45.2% at 2 mV s^−1^ (Figure 4b) that is larger than CdS_surf._‐NiFe‐LDH (16.1%) in Figure S12b (Supporting Information). The role of capacitive contribution of the CdS_inter._‐NiFe‐LDH was increased to 82.2% as the augment of the scanning speed, which is superior to CdS_surf._‐NiFe‐LDH (61.3%), indicating that the redox reaction processes were accelerated because of the interlayer CdS NDs and expanded layer spacing (Figure 4c).

The electrochemical impedance spectroscopy (EIS) plots were performed to investigate the charge transfer ability and OH^−^ ion diffusion of all electrodes (Figure 4d). The internal resistance (*R*_s_) and the charge transfer resistance (*R*_ct_) for the CdS_inter._‐NiFe‐LDH was 0.58 and 0.56 Ω (Table S4, Supporting Information), much smaller than that of CdS_surf._‐NiFe‐LDH (0.97 and 0.81 Ω) due to the lower ion/electron transport limitation after the introduction of interlayer CdS NDs. The equivalent circuit diagram (inset of Figure 4d). Equations 6 and 7 (Seen in Experimental Section) were used to further determine the diffusion coefficient (D) value of electrolyte ion (OH^−^). The *σ* values of CdS_inter._‐NiFe‐LDH (38.02) were much smaller than that of the CdS_surf._‐NiFe‐LDH (85.2), indicating much rapid diffusion rates of OH^−^ (Figure 4e). The corresponding D value of OH^−^ was 1.6 × 10^−8^ cm^2^ s^−1^, much larger than that of the CdS_surf._‐NiFe‐LDH (2.1 × 10^−9^ cm^2^ s^−1^), confirming the superior OH^−^ diffusion and transportation kinetics. According to Equations 8 and 9 (Seen in Experimental Section), the relaxation time constant *τ*_0_ of the CdS_inter._‐NiFe‐LDH was 2.31 ms, and it is significantly smaller than that of the CdS_surf._‐NiFe‐LDH (3.45 ms), further verifying the faster diffusion of the OH^−^ for CdS_inter._‐NiFe‐LDH (Figure 4f). To sum up, the possible mechanism for the enhanced performance of electrode materials can be proposed (Figure 4g). In detail, the redox reaction for CdS_surf._‐NiFe‐LDH mainly occurred in the host lamellar. Although the superficial CdS NDs contribute a variety of oxidation reactions, the reaction rate performance and the interlayer ion flow were still not improved for NiFe‐LDH. On the contrary, for CdS_inter._‐NiFe‐LDH, CdS NDs in the layer can attract more OH^−^ because of the low oxidation potential and easy redox reaction, forming a barren OH^−^ area in the LDH and a rich OH^−^ region in the CdS NDs. Due to the existence of potential difference, a new electric field is generated on the layer between CdS NDs and LDH, which was conducive to the migration of OH^−^ in the charge process and promoted the interlayer reaction to improve the rate performance.

**Figure 5:**
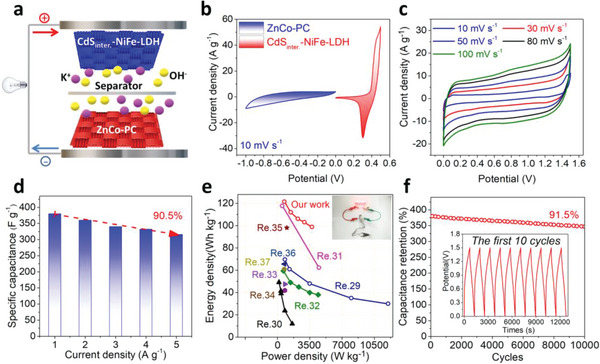
a) The schematic of CdS_inter._‐NiFe‐LDH//ZnCo‐PC device, b) the CV curves of CdS_inter._‐NiFe‐LDH and ZnCo‐PC at 10 mV s^−1^, c) the CV curves of CdS_inter._‐NiFe‐LDH//ZnCo‐PC device at various scanning speed (10–100 mV s^−1^), d) the specific capacitance of CdS_inter._‐NiFe‐LDH//ZnCo‐PC device varies with various current densities, e) the corresponding energy density and power density of CdS_inter._‐NiFe‐LDH//ZnCo‐PC higher than that of other reported ASC device (the illustration display devices lighting the LEDs), f) the cycle performance of CdS_inter._‐NiFe‐LDH//ZnCo‐PC device under 10 000 times (the illustration reveal the first 10 cycles).

We used porous carbon as cathode materials based on ZnCo metal–organic frameworks after high temperature calcination (ZnCo‐PC) for the subsequent assembling SCs device to estimate the potential practical application of the CdS_inter._‐NiFe‐LDH. The SEM image (Figure S13a, Supporting Information) shows that ZnCo‐PC has the loose porous surfaces that can facilitate electrolyte diffusion. XRD patterns reveal that representative peak at 25.37° corresponding to (002) crystal face of graphite was lower than 26° that graphitic carbon has a nice, ordered structure, indicating that the ZnCo‐PC has a better level of graphitization (Figure S13b, Supporting Information). The Raman spectra show that the intensity ratio (I_D_/I_G_) of D band (1340 cm^−1^) and G band (1580 cm^−1^) is 1.32, favoring the conduction of electrons with more defects of C atomic crystal (Figure S13c, Supporting Information). The *C*_s_ of ZnCo‐PC was 320 F g^−1^, as shown in Figure S14 (Supporting Information). The asymmetric SCs(ASC) device was assembled with CdS_inter._‐NiFe‐LDH as anode materials and ZnCo‐PC as the cathode materials in the electrolyte (3.0 m KOH) in **Figure** **5**a (CdS_inter._‐NiFe‐LDH//ZnCo‐PC). The CdS_inter._‐NiFe‐LDH and ZnCo‐PC had stable and compatible voltage window at 10 mV s^−1^ to prove the feasibility for assembling. The mass loading ratio of anode and cathode for assembling ASC device was 0.19 based on Equation 10 (Seen in Experimental Section) in Figure 5b. By measuring the CV curves at the same scanning speed of 50 mV s^−1^ under various voltage windows, we determined the voltage window of 1.5 V for ASC device (Figure S15, Supporting Information). The shape of the CV curves maintained invariant under the same voltage window with increased scanning speed (Figure 5c). The ultra‐high capacitance of the ASC device was 380 F g^−1^ and the capacitance retention with a fivefold increase in current density of 90.5% (Figure 5d). According to Equations 11 and 12 (Seen in Experimental Section), the CdS_inter._‐NiFe‐LDH//ZnCo‐PC device reveals a maximum energy density of 121.56 Wh kg^−1^ at a power density of 754.5 W kg^−1^ and still reached 98.75 Wh kg^−1^ at a high power density of 3742.0 W kg^−1^ (Figure 5e), higher than that of other reported LDH‐based ASC (Table S5, Supporting Information).^[^ ^29^, ^30^, ^31^, ^32^, ^33^, ^34^, ^35^, ^36^, ^37^ ^]^ The prepared button‐type devices can enable the LEDs in parallel (illustration of Figure 5e). According to the GCD curves at 1 A g^−1^, the CdS_inter._‐NiFe‐LDH//ZnCo‐PC device kept 91.5% of original capacitance after 10 000 cycles (Figure 5f). As compared to the GCD curves for the first 10 cycles, the device represents excellent stability and reversibility. The EIS of the ASC devices after 10 000 cycles shows that the slope of the curve did not change significantly at the low frequency, indicating the outstanding electrochemical stability of ASC device (Figure S16, Supporting Information).

## 3 Conclusion

In summary, we develop a general strategy to modify interlayers of NiFe‐LDH with CdS NDs via electrochemical cycling and vulcanization at low temperature. As prepared CdS_inter._‐NiFe‐LDH electrode possesses high capacitance (3330.0 F g^−1^ at 1 A g^−1^) and rate performance (90.9% at 10 A g^−1^), which is higher than the counterpart with CdS NDs on the surface of NiFe‐LDH (1966.6 F g^−1^ at 1 A g^−1^and 48.0% at 10 A g^−1^). Such interlayer NDs modification leads to an outstanding ECSA (601 mF cm^−2^) and transfer efficiency of OH^−^ (1.6 × 10^−8^ cm^2^ s^−1^) by the fast diffusion of electrolyte ions between layers. More importantly, the assembled CdS_inter._‐NiFe‐LDH//ZnCo‐PC device reveals good energy density of 121.56 Wh kg^−1^ and a superior capacitance retention of 91.5% after 10 000 cycles. The excellent performance results from that the Cd^2+^ cation embedded in the layers of NiFe‐LDH and the subsequently formed CdS NDs at the low temperature, which can enlarge the layer spacing, anchor highly active units on inner surface, and maintain the original lamellar structure of LDH. Therefore, it is a significant strategy that introducing NDs between LDH layers to break through the bottleneck of the low flow efficiency of OH^−^ between LDH layers.

## 4 Experimental Section

### Preparation of NiFe‐LDH

NiCl_2_·6H_2_O (3 mmol), FeCl_3_·6H_2_O (1 mmol), and NH_4_F (12 mmol) were diffused into 30 mL deionized (DI) water to obtain a homogeneous solution as A solution. Then, 19.2 mmol urea was added into 30 mL DI water as B solution. Mixed A and B were dissolved for 10 min. The above suspension was shifted to a 100 mL autoclave, and a piece of carbon cloth (1 × 3 cm^2^) was placed in it at 120 °C for 5 h. The sample was collected and warmed at 60 °C for 12 h. The even mass loading of NiFe‐LDH/CC was 1.5 mg cm^−2^.

### Preparation of Cd^2+^/NiFe‐LDH

Cd^2+^ intercalation was prepared by adopting a CHI660E workstation in a three‐electrode electrochemical system, in which the NiFe‐LDH as work electrode, saturated calomel electrode (SCE) as reference electrode, and platinum as counter electrode. The cadmium source was selected as 1 m CdCl_2_ solution. Cyclic voltammetry tests were performed in the voltage range 0–1.0 V at scanning speeds of 10–100 mV s^−1^. The sample was washed with DI water and warmed in a 60 °C vacuum drying oven.

### Preparation of CdS_inter._‐NiFe‐LDH

Na_2_S·9H_2_O (1 g) was added to 40 mL DI water to stir for 10 min. The above carbon cloth was soaked in a solution for 0.5, 1, 1.5, and 2 h at diverse temperature (0, 5, 10, and 15 °C). The resulting sample was washed three times with ethanol and warmed in vacuum drying oven at 60 °C until dried. The CdS_surf._‐NiFe‐LDH was synthesized by immersion in CdCl_2_ solution without electrochemical cycle. Other preparation conditions were the same as CdS_inter._‐NiFe‐LDH. In order to further optimize the electrochemical performance of the electrode, the content of CdS in CdS_inter._‐NiFe‐LDH was further optimized with different CdCl_2_ solution concentrations (0.1–5 m), noted as CdS_inter._‐NiFe‐LDH‐0.1∼ CdS_inter._‐NiFe‐LDH‐5.

### Preparation of ZnCo‐PC

Zn(NO_3_)_2_·6H_2_O (0.3 mmol) and Co(NO_3_)_2_·6H_2_O (0.6 mmol) were dissolved into 20 mL DI water stirring for 10 min to form a clean solution as A solution. 2‐Methylimidazole (2MI) (7.5 mmol) was stirred to 20 mL DI water for 10 min as B solution. The B solution was quickly poured to A solution with stirring 10 min, and then a piece of carbon cloth was immersed. The obtained carbon cloth was rinsed with DI water and ethanol and warmed for 24 h. The porcelain boat with carbon cloth was placed in a tubular furnace under argon gas and heated for 2 h at a rate of 2 °C min^−1^. Samples were collected after cooling.

### Characterization

The SEM image of samples was obtained using JSM‐7500F. TEM was adopted to investigate morphology and structure of electrodes on JEOL JEM‐2010 at the accelerating voltage of 200 kV. XRD curves were surveyed on a shimazu‐6100 powder, Japan with Cu K*α* radiation (1.5418 Å). The chemical valence and elemental surveying were researched by XPS on Escalab 250Xi. The atomic ratios of samples were conducted by ICP‐OES tests. The AFM was performed in ambient conditions using Easyscan2 Flex.

### Electrochemical Measurement

The three‐electrode system was employed to survey electrochemical performances of the electrodes on a CHI660e workstation, which was used with Pt plate counter electrode and Hg/HgO reference electrode (with a salt bridge of 3 m KOH aqueous solution). The CV at diverse scanning speed (2–50 mV s^−1^), GCD at diverse current densities (1–5 A g^−1^), and EIS plots (1.0 Hz to 10^5^ Hz) of all electrodes were obtained in 3 m KOH electrolyte.

The following formula to obtain C_s_ (F g^−1^) by the GCD curves:

(1) $$C_{s}=\mathrm{it}/m\Delta V$$

Here, *i*, *t*, *m*, and Δ*V* are the current (*A*), discharge time (*s*), quality loading of electrode (*g*), and potential difference (*V*), respectively.

The double layer area specific capacitance (*C*_DL_) abided by the following equation:

(2) $$I_{\mathrm{DL}}=C_{\mathrm{DL}}v$$

*I*_DL_ and *v* are capacitance current and scanning rate, respectively.

The equation was to calculate electrochemical active surface area (ECSA) of electrodes:

(3) $$\mathrm{ECSA}=C_{\mathrm{DL}}/C_{S}$$

*C*_s_ is constant of 0.04 mF cm^−2^ in an alkaline electrolyte.

Generally, the current response (*I*) and scan rates (*v*) observe the following equation:

(4) $$I=av^{b}$$

(5) $$I\left(V\right)=k_{1}v+k_{2}v^{1/2}$$

*I* is peak current densities; *k*_1_v is capacitive contribution, and *k*_2_v^1/2^ is the diffusion‐controlled contribution, respectively.

The following equation was to calculate the diffusion coefficient value (D) of OH^−^:

(6) $$D=\frac{R^{2}T^{2}}{2A^{2}n^{4}F^{4}C^{2}{R\sigma }^{2}}$$

(7) $$Z^{\prime }=R_{s}+R_{\mathrm{ct}}+\sigma \omega^{-1/2}$$

*R* (J mol^−1^ K^−1^), *T* (K), *A* (cm^−2^), n, *F* (C mol^−1^), *C* (L mol^−1^), *σ*, and *ω* (Hz) are the gas constant, absolute temperature, reaction area of electrode, quantity of transferred electrons, Faraday constant, concentration of electrolyte, Warburg diffusion coefficient, and angular frequency, respectively.

The following equation to obtain the complex modality of capacitance:

(8) $$C^{\prime }\left(\omega \right)=\frac{-Z^{\prime \prime }\left(\omega \right)}{\omega {\left|Z\left(\omega \right)\right|}^{2}}$$

(9) $$C^{\prime \prime }\left(\omega \right)=\frac{Z^{\prime }\left(\omega \right)}{\omega {\left|Z\left(\omega \right)\right|}^{2}}$$

*C*′(*ω*) and (*ω*), respectively, were real and imaginary part of capacitance; *Z*′′(*ω*) was imaginary part of the impedance.

The ASC device was equipped by CdS_inter._‐NiFe‐LDH as anode materials, ZnCo‐PC as cathode materials, and JIAO JIE qualitative filter paper soaked in 3 MKOH solution as separator.

According to the charge balance between anode and cathode, the mass loading (Δ*m*) of cathode was obtained by the following formula:

(10) $$m_{+}/m_{-}=C_{a-}\Delta V_{-}/C_{a+}\Delta V_{+}$$

The power density (P, W kg^−1^) and energy density (E, Wh kg^−1^) of the device were acquired by integral equations:

(11) P=3600E/Δt

(12) $$E=0.5C\Delta V^{2/3.6}$$

## Conflict of Interest

The authors declare no conflict of interest.

## Supporting information

Supporting Information

## Data Availability

The data that support the findings of this study are available from the corresponding author upon reasonable request.
