# Supplementary material for: Interlayer Nano‐Dots Induced High‐Rate Supercapacitors
Source: Adv Sci (Weinh). 2023 Jun 4;10(23):2301398. doi: 10.1002/advs.202301398 (PMC10427355; doi:10.1002/advs.202301398)
Supplement: Supplementary file 1 — Supporting Information [file ADVS-10-2301398-s001.pdf]

ADVANCED SCIENCE
Open Access

Supporting Information

for *Adv. Sci.*, DOI 10.1002/advs.202301398

Interlayer Nano-Dots Induced High-Rate Supercapacitors

Chunyan Li, Xinkun Wang, Dongge Ma, Yan Yan, Pengwei Huo* and Qingjun Yang**

Interlayer Nano-Dots Induced High-Rate Supercapacitors

Chunyan Li[a,b], Xinkun Wang[a], Dongge Ma[c], Yan Yan*[b], Pengwei Huo*[b], Qingjun Yang*[b]

a Research Center of Fluid Machinery Engineering and Technology, Jiangsu University, Zhenjiang 212013, PR China

b School of Chemistry and Chemical Engineering, Jiangsu University, Zhenjiang 212013, PR China

c Department of Chemistry, College of Chemistry and Materials Engineering, Beijing Technology and Business University, Beijing 100048, PR China

*Corresponding author: Prof. Yan Yan

E-mail address: dgy5212004@163.com

*Corresponding author: Prof. Pengwei Huo

E-mail address: huopw@ujs.edu.cn

*Corresponding author: Dr. Qingjun Yang

E-mail address: 18796023937@163.com

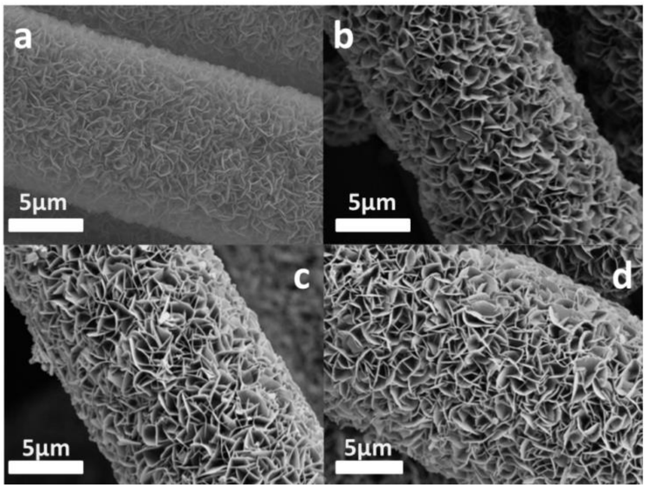

Fig. S1. The SEM images of **(a)** NiFe-LDH, **(b)** Cd^{2+}/NiFe-LDH, **(c)** $CdS_{inter.}$-NiFe-LDH and **(d)** $CdS_{surf.}$-NiFe-LDH.

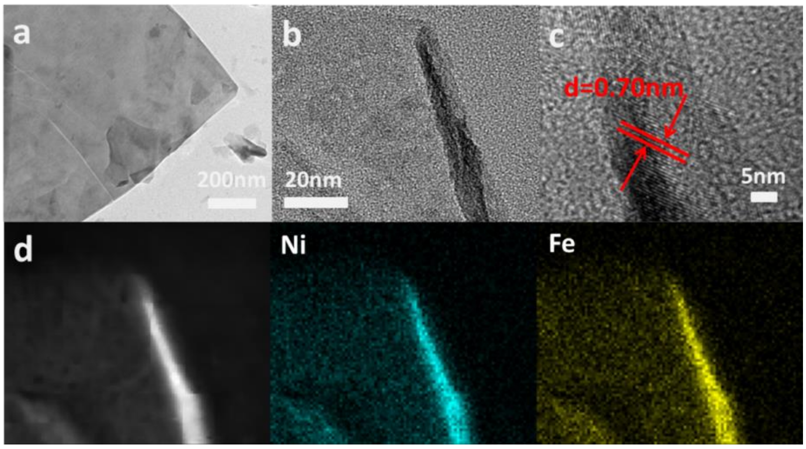

Fig. S2. The TEM image of **(a)** NiFe-LDH, **(b)** the HRTEM image of NiFe-LDH, **(c)** the magnifying HRTEM image of NiFe-LDH and **(d)** the element mapping of NiFe-LDH.

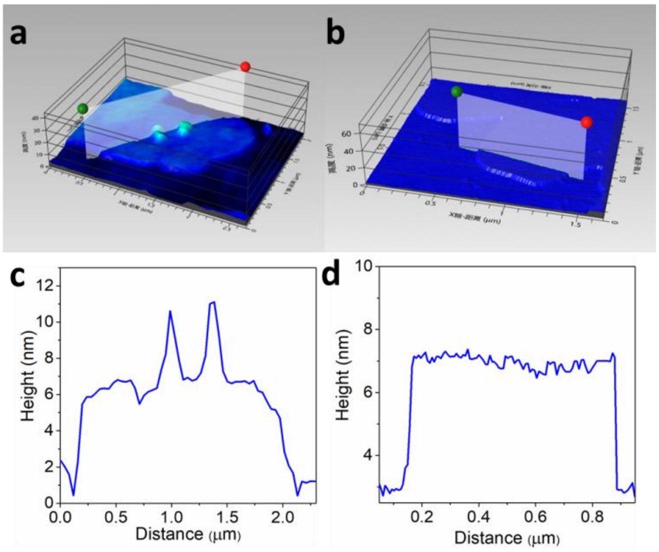

Fig. S3. The 3D AFM images of **(a)** $CdS_{surf.}$-NiFe-LDH and **(b)** $CdS_{inter.}$-NiFe-LDH. The height profiles of **(c)** $CdS_{surf.}$-NiFe-LDH and **(d)** $CdS_{inter.}$-NiFe-LDH.

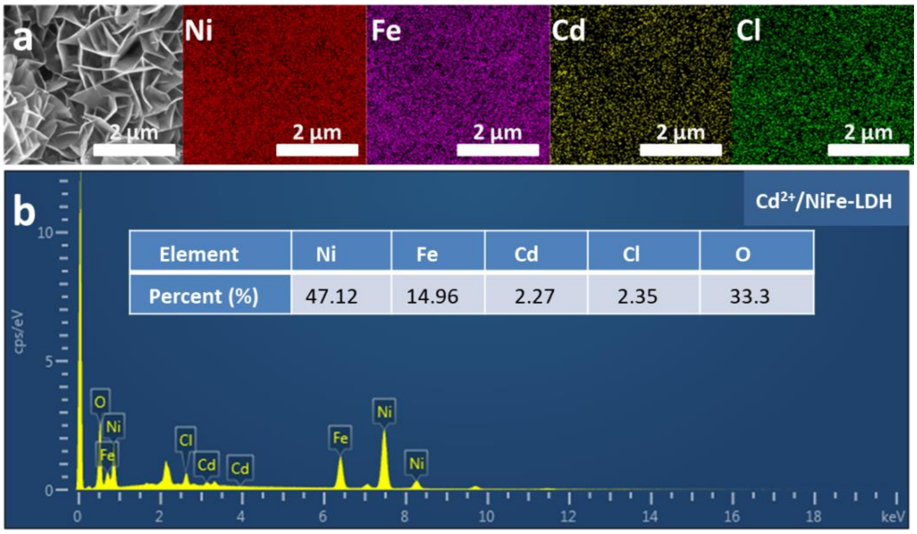

Fig.S4 The element mapping of **(a)** Cd^{2+}/NiFe-LDH by electrochemical cycling and **(b)** The Ni/Fe/Cd/Cl/O atomic ratios in Cd^{2+}/NiFe-LDH by EDS.

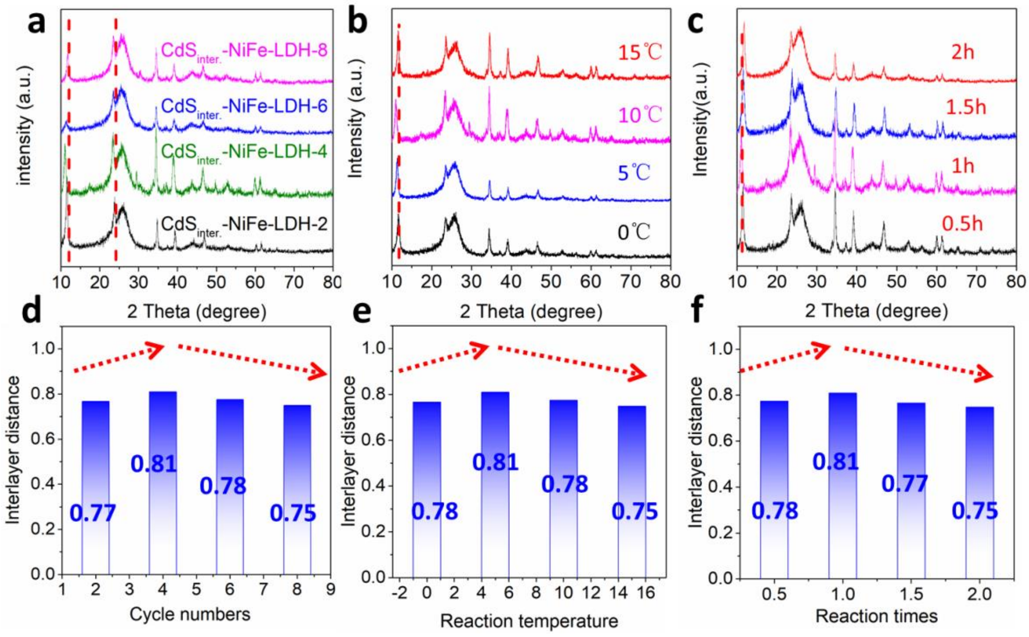

Fig. S5. (a) The XRD patterns of $CdS_{inter.}$-NiFe-LDH at different cycle numbers, **(b)** the XRD patterns of $CdS_{inter.}$-NiFe-LDH at different reaction temperature, **(c)** the XRD patterns of $CdS_{inter.}$-NiFe-LDH at different reaction times, **(d)** the layer distance of $CdS_{inter.}$-NiFe-LDH varies with cycle number from 2 to 8, **(e)** the layer distance of $CdS_{inter.}$-NiFe-LDH varies with reaction temperature from 0 to 15 ℃, **(f)** the layer distance of $CdS_{inter.}$-NiFe-LDH varies with reaction temperature from 0.5 to 2 h.

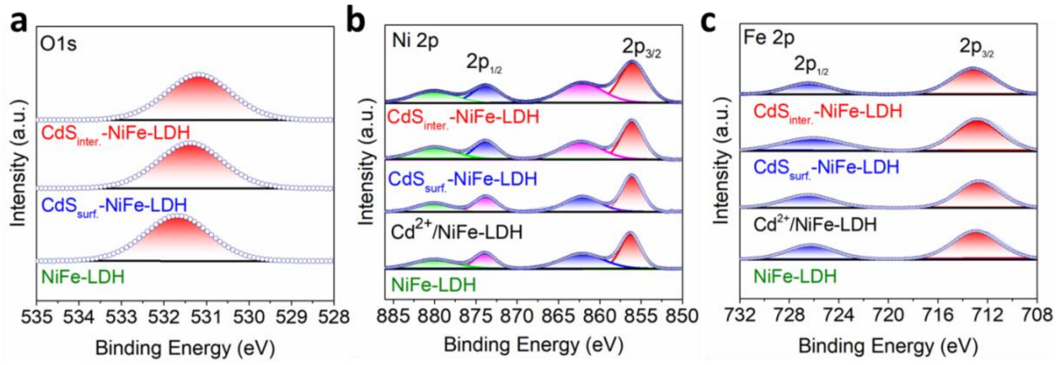

Fig. S6. The XPS spectra of **(a)** O 1s, **(b)** Ni 2p and **(c)** Fe 2p orbits of $CdS_{inter.}$-NiFe-LDH.

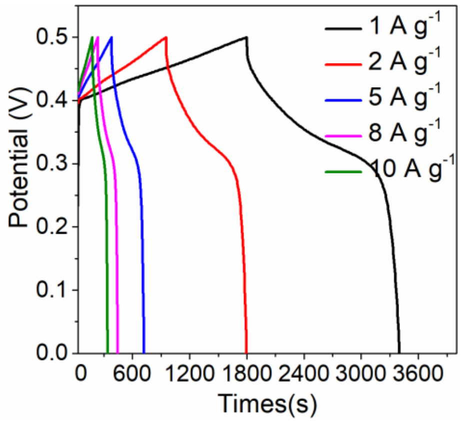

Fig. S7. The GCD curves of $CdS_{inter.}$-NiFe-LDH at different current densities under the voltage window of 0 - 0.5 V.

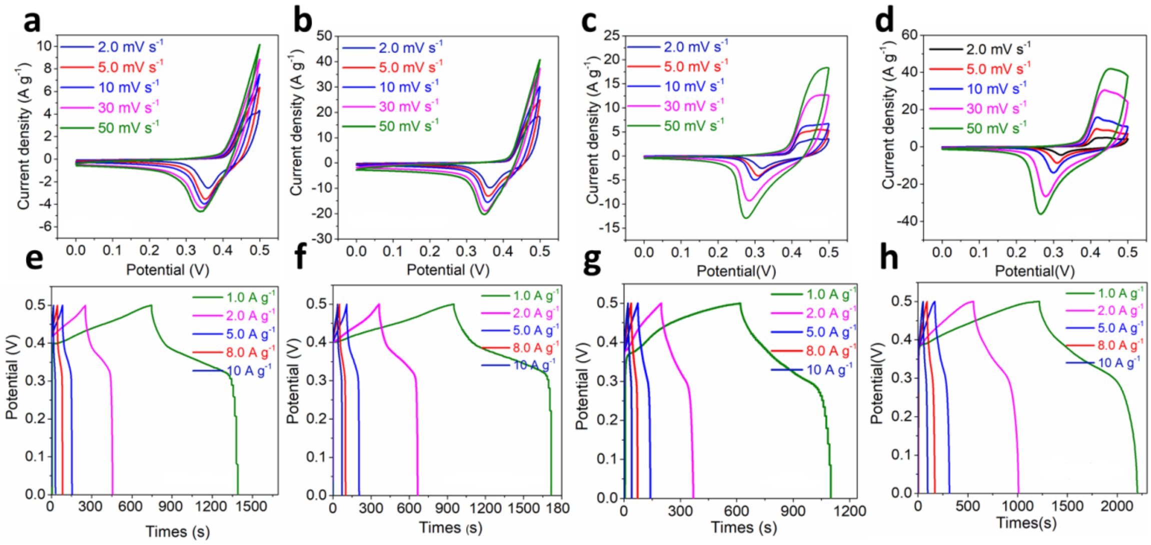

Fig. S8. (a) the CV curves at different scan rates and **(e)** the GCD curves at different current densities under the voltage window of 0 - 0.5 V for NiFe-LDH, **(b)** the CV curves at different scan rates and **(f)** the GCD curves at different current densities under the voltage window of 0 - 0.5 V for Cd^{2+}/NiFe-LDH, **(c)** the CV curves at different scan rates and **(g)** the GCD curves at different current densities under the voltage window of 0 - 0.5 V for CdS, **(d)** the CV curves at different scan rates and **(h)** the GCD curves at different current densities under the voltage window of 0 - 0.5 V for $CdS_{surf.}$-NiFe-LDH.

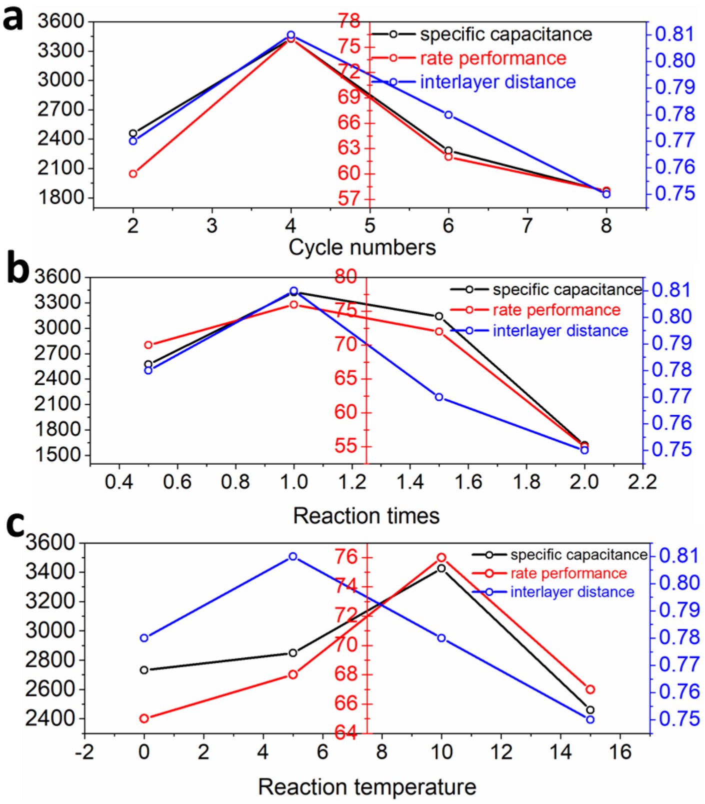

Fig. S9. (a) the specific capacitance, rate performance and layer distance of $CdS_{inter.}$-NiFe-LDH varies with cycle numbers, **(b)** the specific capacitance, rate performance and layer distance of $CdS_{inter.}$-NiFe-LDH varies with reaction times, **(c)** the specific capacitance, rate performance and layer distance of $CdS_{inter.}$-NiFe-LDH varies with reaction temperature.

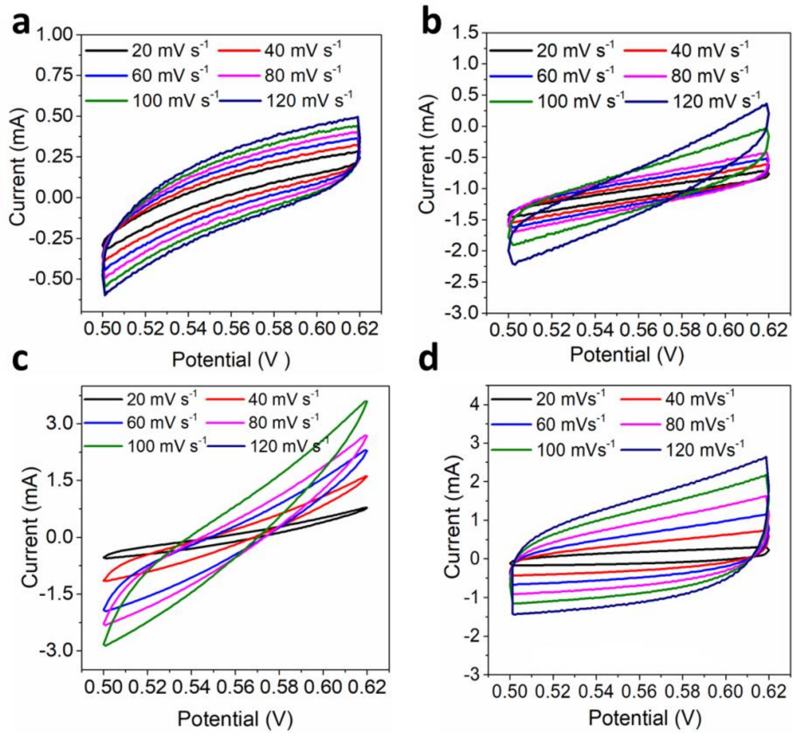

Fig. S10. CV curves of **(a)** NiFe-LDH, **(b)** CdS, **(c)** $CdS_{surf.}$-NiFe-LDH and $CdS_{inter.}$-NiFe-LDH at different scan rates from 20 to 120 mV s^{-1}.

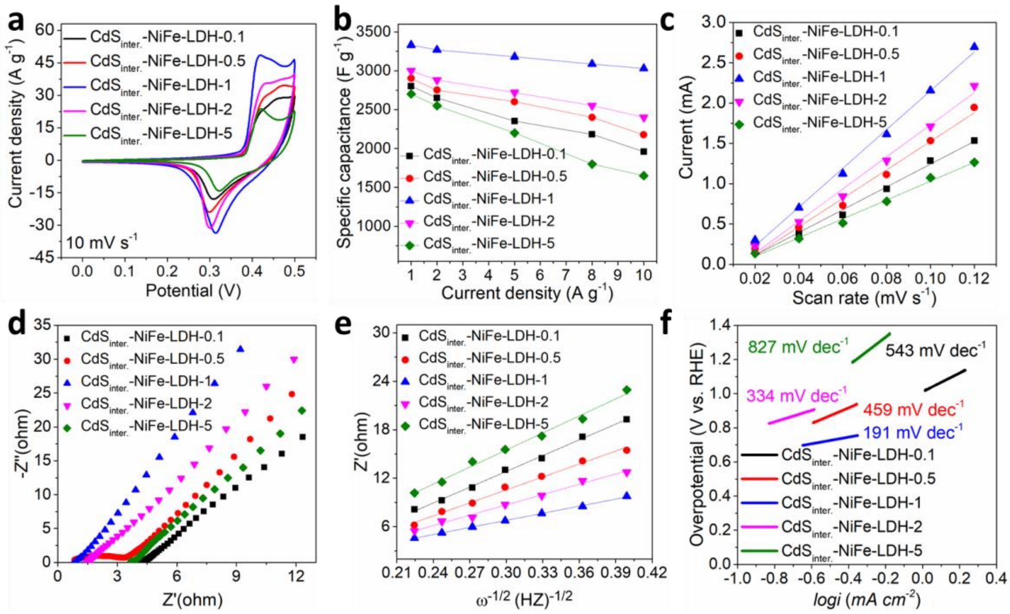

Fig. S11. (a) the CV curves of $CdS_{inter.}$-NiFe-LDH with different content of Cd^{2+} (0.1, 0.5, 1, 2 and 5); **(b)** the specific capacitance of $CdS_{inter.}$-NiFe-LDH with different content of Cd^{2+} (0.1, 0.5, 1, 2 and 5) varies with various current densities (1 - 10 A g^{-1}); **(c)** the capacitive current (I_{DL}) at 0.56 V varies with scanning speed for $CdS_{inter.}$-NiFe-LDH with different content of Cd^{2+} (0.1, 0.5, 1, 2 and 5); **(d)** the EIS plots of $CdS_{inter.}$-NiFe-LDH with different content of Cd^{2+} (0.1, 0.5, 1, 2 and 5); **(e)** The Z' of $CdS_{inter.}$-NiFe-LDH with different content of Cd^{2+} (0.1, 0.5, 1, 2 and 5) varies with $\omega^{-1/2}$ at low frequency section; **(f)** Tafel graphs of $CdS_{inter.}$-NiFe-LDH with different content of CdS (0.1, 0.5, 1, 2 and 5).

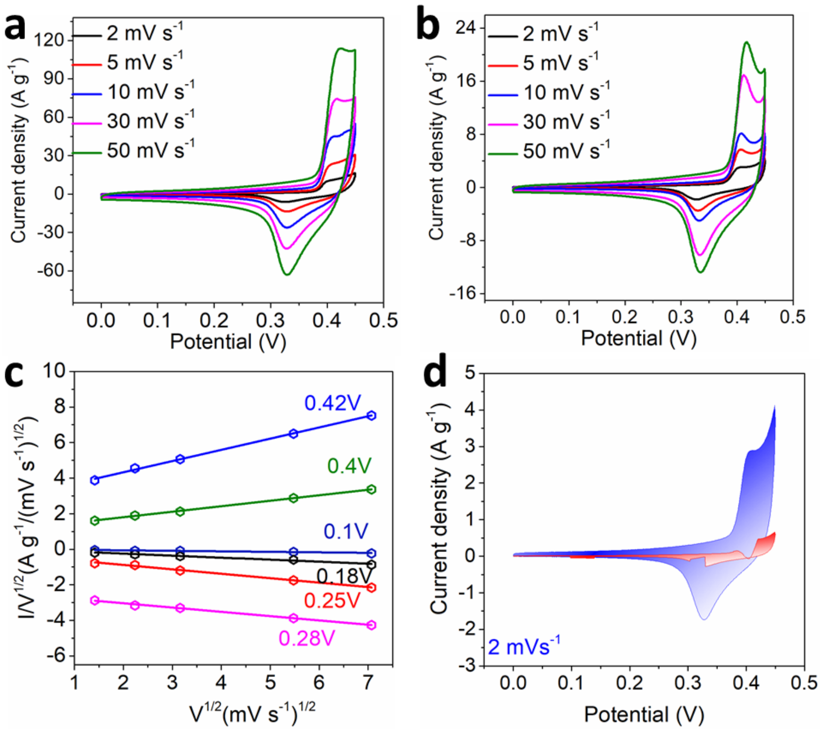

Fig. S12. CV curves of **(a)** $CdS_{inter.}$-NiFe-LDH and **(b)** $CdS_{surf.}$-NiFe-LDH, **(c)** The linear relationship between $v^{-1/2}$ and $i/v^{-1/2}$ to calculate k_1 and k_2, **(d)** Voltammetric response for the surfaced $CdS_{surf.}$-NiFe-LDH at a scan rate of 2 mV s^{-1}.

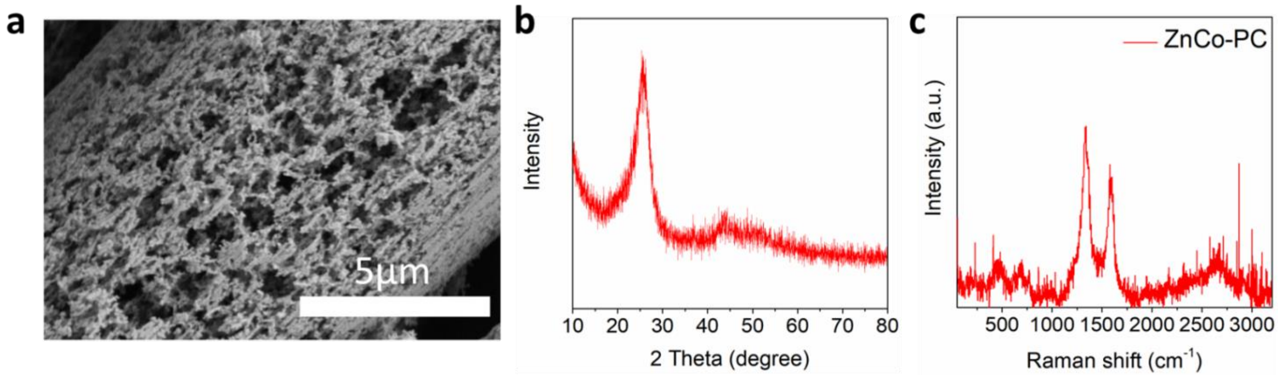

Fig. S13. (a) The SEM image, **(b)** XRD pattern and **(c)** the Raman shift of ZnCo-PC.

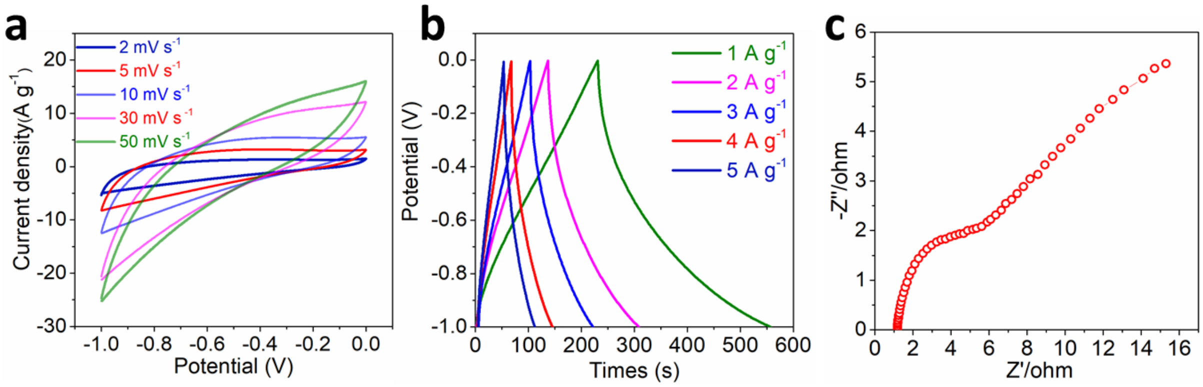

Fig. S14. (a) the CV curves of ZnCo-PC at different scan rates of 2 - 50 mV s^{-1}, **(b)** the GCD curves of ZnCo-PC at different current densities of 1 - 5 A g^{-1}, **(c)** the EIS plot of ZnCo-PC.

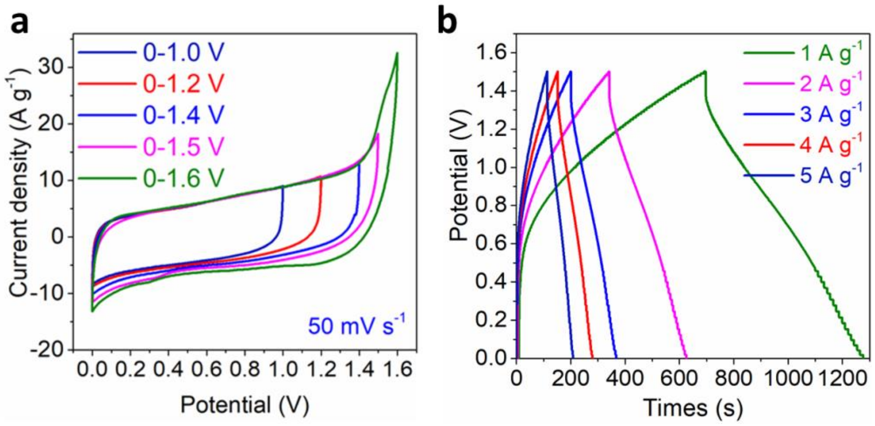

Fig. S15. **(a)** The CV curves of $CdS_{inter.}$-NiFe-LDH//ZnCo-PC device under different voltage window at a scan rate of 50 mV s^{-1}, **(b)** the GCD curves of $CdS_{inter.}$-NiFe-LDH//ZnCo-PC device at different current densities of 1 - 5 A g^{-1}.

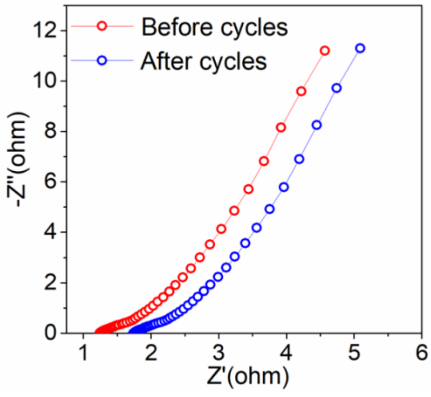

Fig. S16. EIS plots of cathode of $CdS_{inter.}$-NiFe-LDH//ZnCo-PC device before and after cycles.

As the vulcanization time increased to 2 h, the NDs quantity of $CdS_{inter.}$-NiFe-LDH gradually decreased (**Fig. S17a - b**). On the contrary, the NDs quantity increased gradually when the vulcanization temperature increased to room temperature (**Fig. S17d - f**).

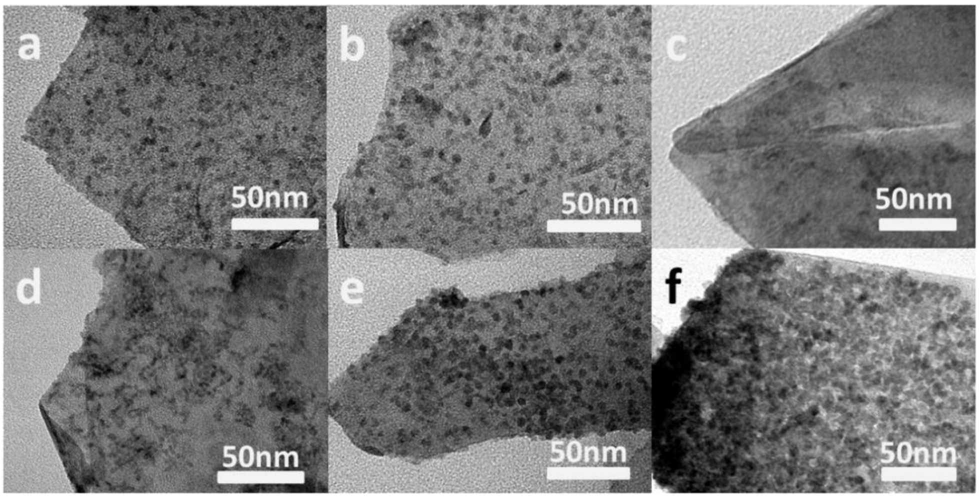

Fig. S17. The TEM images of **(a)** $CdS_{inter.}$-NiFe-LDH electrode at the reaction time of 0.5 h, **(b)** $CdS_{inter.}$-NiFe-LDH at the reaction time of 1.5 h, **(c)** $CdS_{inter.}$-NiFe-LDH at the reaction time of 2 h, **(d)** $CdS_{inter.}$-NiFe-LDH at the reaction temperature of 0 ℃, **(e)** $CdS_{inter.}$-NiFe-LDH at reaction temperature of 5 ℃, and **(f)** $CdS_{inter.}$-NiFe-LDH at reaction temperature of 15 ℃.

The CV and GCD curves of $CdS_{inter.}$-NiFe-LDH with different cycle numbers, reaction times and reaction temperature were showed in **Fig. S18, Fig. S19** and **Fig. S20**.

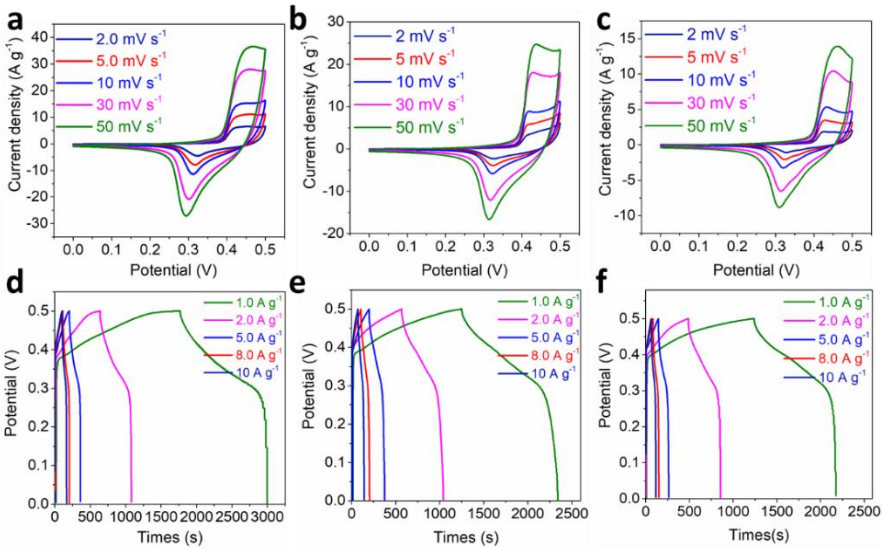

Fig. S18. (a) the CV curves at different scan rates and **(d)** the GCD curves at different current densities of $CdS_{inter.}$-NiFe-LDH with the cycle numbers of 2 under the voltage window of 0 - 0.5 V, **(b)** the CV curves at different scan rates and **(e)** the GCD curves at different current densities of $CdS_{inter.}$-NiFe-LDH with the cycle numbers of 6 under the voltage window of 0 - 0.5 V, **(c)** the CV curves at different scan rates and **(f)** the GCD curves at different current densities of $CdS_{inter.}$-NiFe-LDH with the cycle numbers of 8 under the voltage window of 0 - 0.5 V.

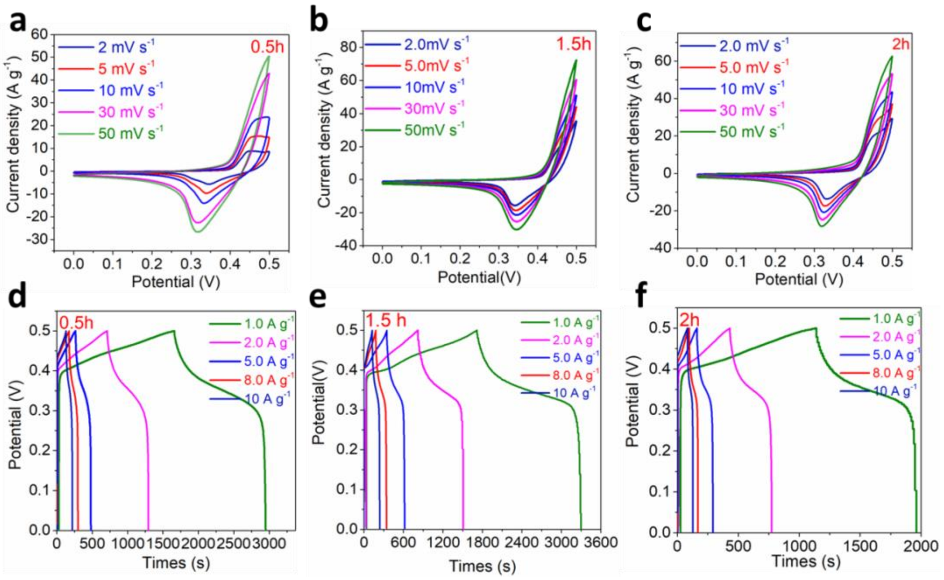

Fig. S19. **(a)** the CV curves at different scan rates and **(d)** the GCD curves at different current densities of $CdS_{inter.}$-NiFe-LDH with the reaction time of 0.5 h under the voltage window of 0 - 0.5 V, **(b)** the CV curves at different scan rates and **(e)** the GCD curves at different current densities of $CdS_{inter.}$-NiFe-LDH with the reaction time of 1.5 h under the voltage window of 0 - 0.5 V, **(c)** the CV curves at different scan rates and **(f)** the GCD curves at different current densities of $CdS_{inter.}$-NiFe-LDH with the reaction time of 2 h under the voltage window of 0 - 0.5 V.

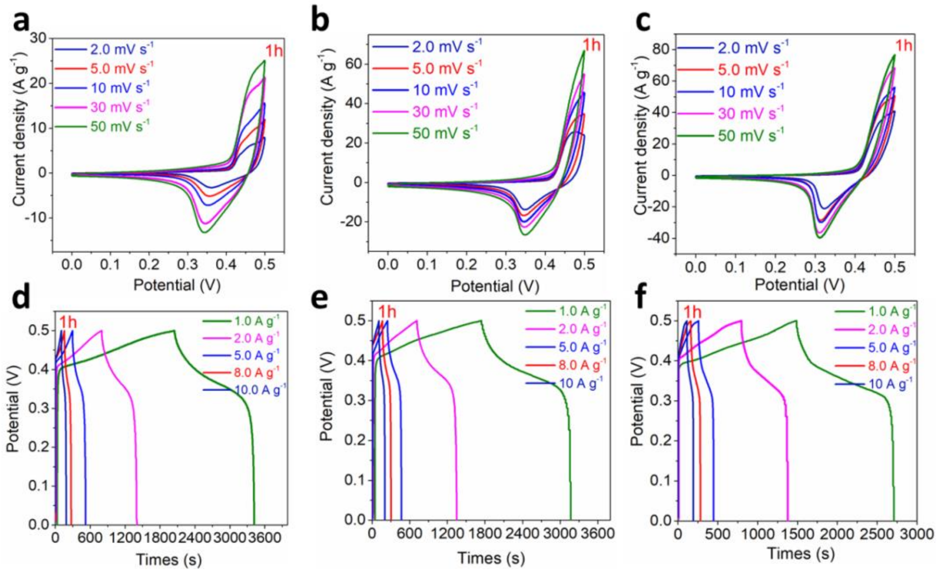

Fig. S20. **(a)** the CV curves at different scan rates and **(d)** GCD curves at different current densities of $CdS_{inter.}$-NiFe-LDH with the reaction temperature of 0 ℃ under the voltage window of 0 - 0.5 V, **(b)** the CV curves at different scan rates and **(e)** the GCD curves at different current densities of $CdS_{inter.}$-NiFe-LDH with the reaction temperature of 5 ℃ under the voltage window of 0 - 0.5 V, **(c)** the CV curves at different scan rates and **(f)** the GCD curves at different current densities of $CdS_{inter.}$-NiFe-LDH with the reaction temperature of 15 ℃ under the voltage window of 0 - 0.5 V.

Table S1. The Ni/Fe/Cd/S atomic ratios in $CdS_{inter.}$-NiFe-LDH and $CdS_{surf.}$-NiFe-LDH by ICP-OES.

Element	Ni	Fe	Cd	S
$CdS_{inter.}$-NiFe-LDH	8.5648	3.6555	5.2426	1.0482
$CdS_{surf.}$-NiFe-LDH	10.621	4.325	6.463	2.106

Table S2 The electrochemical performance of $CdS_{inter.}$-NiFe-LDH compared with other works.

**Electrode materials**	**Specific capacitance**	**Rate performance**	**Cycle performance**	**Refs.**
NiS@Sr-FeOH/CC	1553 F g^{-1} at 1A g^{-1}	76% at 5 A g^{-1}	87% (5000 cycles)	**[1]**
NiMn-LDH/hrGO	302 C g^{-1} at 1A g^{-1}	64% at 40 mV s^{-1}	80.5% (2000 cycles)	**[2]**
CoG@$Ni_3(NO_3)_2{\cdot}(OH)_4$	280 mAh g^{-1} at 1A g^{-1}	77% at 10 A g^{-1}	81.8% (5000 cycles)	**[3]**
CNS/MnO_2/$NiCo_2O_4$	1254 F g^{-1} at 0.25A g^{-1}	60.1% at 2 A g^{-1}	81.9% (5000 cycles)	**[4]**
Co-Ni_3S_4-NiS/Ni	1940 F g^{-1} at 1A g^{-1}	86.3% at 10 A g^{-1}	79% (3000 cycles)	**[5]**
$NiCoSe_4$/N-rGO	120 mAh g^{-1} at 1 A g^{-1}	80% at 10 A g^{-1}	83% (3000 cycles)	**[6]**
**$CdS_{inter.}$-NiFe-LDH**	**3330 F g^{-1} at 1 A g^{-1}**	**90.9% at 10 A g^{-1}**	**88.2% (5,000 cycles)**	**Our work**

Table S3. The Ni/Fe/Cd/S atomic ratios in $CdS_{inter.}$-NiFe-LDH with different CdS content by ICP test.

Elements	Ni	Fe	Cd	S
$CdS_{inter.}$-NiFe-LDH-0.1	6.156	2.152	0.812	0.221
$CdS_{inter.}$-NiFe-LDH-0.5	6.913	2.789	1.554	1.015
$CdS_{inter.}$-NiFe-LDH-1	6.452	3.235	2.151	1.513
$CdS_{surf.}$-NiFe-LDH-2	7.121	3.954	3.152	2.561
$CdS_{inter.}$-NiFe-LDH-5	8.912	4.023	3.713	2.963

Table S4 EIS parameters obtained by fitting EIS spectra of $CdS_{inter.}$-NiFe-DLH to a suitable equivalent circuit.

Parameter	$CdS_{inter.}$-NiFe-LDH	$CdS_{surf.}$-NiFe-LDH	NiFe-LDH	CdS
R_s/Ω	0.58	0.97	1.118	0.7137
R_{ct}/Ω	0.56	0.81	1.052	0.6992

Table S5 The energy density and power density of $CdS_{inter.}$-NiFe-LDH//ZnCo-PC device compared with other works.

**ASC device**	**Energy density**	**Power density**
Co_3O_4/MoCo/LDH/NF//AC	67.62 W h kg^{-1}	800 W kg^{-1}
NiZn-LDH@$NiCoSe_2$//AC	49.2 W h kg^{-1}	160 W kg^{-1}
CQDs/NiCo-LDHs@CC//AC	110 W h kg^{-1}	800 W kg^{-1}
AHAS	59.37 W h kg^{-1}	640 W kg^{-1}
COH@NF-LDH/CF//AC	65.56 W h kg^{-1}	750 W kg^{-1}
NiCo-LDH-CQDs-20//AC	47.47 W h kg^{-1}	800 W kg^{-1}
$NiCoMoS_{2/1}$@LDH//AC	41.91 W h kg^{-1}	800 W kg^{-1}
$CuBr_2$@NCC-LDH/CF//AC	98 W h kg^{-1}	1013 W kg^{-1}
Ni_3S_2 @NMV-L/rGO-15//AC	60 W h kg^{-1}	849 W kg^{-1}
$CdS_{inter.}$-NiFe-LDH//ZnCo-PC	121.56 Wh kg^{-1}	754.5 W kg^{-1}

Reference

[1] A. Rajapriya, S. Keerthana, C. Viswanathan, N. Ponpandian, J. Energy Storage **2022**, 53, 7756.

[2] W. Yan, Y. Zhang, T. Zeng, Y. Zhang, Q. Wan, N. Yang, J. Energy Storage **2022**, 52, 11256.

[3] T. Cui, S. Wu, S. Zhou, Q. Feng, X. Xu, H. Zhao, Q. Su, Y. Wang, X. Zhao, Q. Yang, J. Energy Storage **2022**, 52, 100021.

[4] X. Hong, C. Deng, X. Wang, W. Dong, B. Liang, J. Energy Storage **2022**, 53, 5679.

[5] Y. Wang, X. He, X. Chen, Y. Zhang, F. Li, Y. Zhou, C. Meng, Appl. Surf. Sci. **2022**, 596, 153600.

[6] Q. Yang, Q. Feng, X. Xu, Y. Liu, X. Yang, F. Yang, J. Li, H. Zhan, Q. Wang, S. Wu, Nanotechnology **2022**, 33, 5623.
